# Optical coherence tomography angiography and fundus autofluorescence in the eyes with choroideremia

**DOI:** 10.1136/bcr-2016-217682

**Published:** 2017-01-06

**Authors:** Maki Kato, Ichiro Maruko, Hideki Koizumi, Tomohiro Iida

**Affiliations:** Department of Ophthalmology, Tokyo Women's Medical University School of Medicine, Tokyo, Japan

## Abstract

A 65-year-old man with presumed choroideremia with preserved central vision was examined by fundus autofluorescence (FAF) and optical coherence tomography angiography (OCTA). FAF showed an isolated area of hyperautofluorescence that involved the fovea. Although the choroid capillary slab of the OCTA showed the medium and large choroidal vessels inferior to the area of retinal pigment epithelium (RPE) atrophy, the choriocapillaris was visible in a relatively wider area than the hyperautofluorescent area in the FAF images. FAF and OCTA images allowed us to detect damage of the RPE before the choriocapillaris atrophy in a case of presumed choroideremia with preserved central vision.

## Background

Since choroideremia has received much attention as a disease that might be successfully treated by gene therapy, it has become important to evaluate the physiology and morphology of the retina and choroid using the various retinal imaging techniques. Fundus autofluorescence (FAF) is a relatively new method of evaluating the retinal pigment epithelium (RPE) function. It should be possible to obtain useful information on eyes with choroideremia because this disease is associated with the RPE atrophy. Optical coherence tomography angiography (OCTA) is another new technique that can detect the retinal blood vessel patterns in en face images. However, there are few studies on the choroidal blood pattern using OCTA because the vessels are not visible in normal individuals. However, a recent report shows the choroidal vessels in the OCTA images of eyes with RPE atrophy. OCTA might be helpful to evaluate the choroidal circulation associated with the RPE function in choroideremia.

## Case presentation

**Figure 1:**
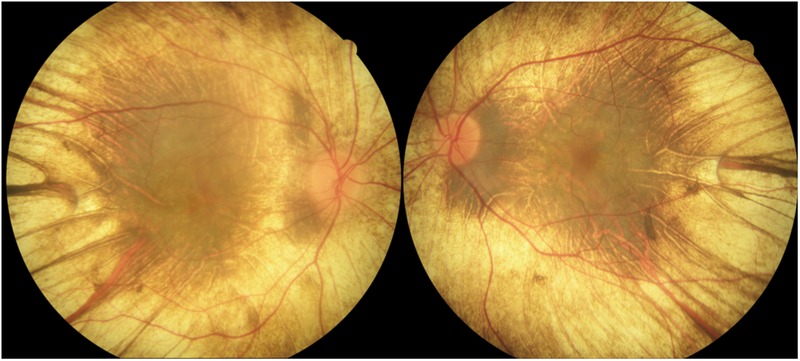
Fundus photographs of both eyes of a patient with choroideremia. Medium and large choroidal vessels can be seen throughout the fundus due to atrophy of the RPE and choriocapillaris. The sclera can be seen except in the area of pigment aggregation in the macular area. RPE, retinal pigment epithelium.

**Figure 2:**
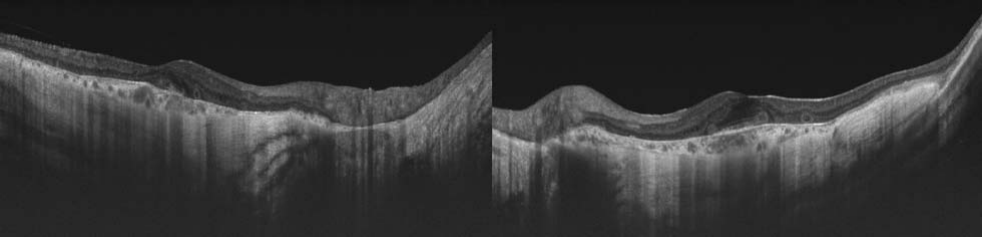
Horizontal optical coherence tomographic images of both eyes. The foveal depression is preserved in both eyes; however, the outer retina is thin except in the foveal area. The ellipsoid zone is indistinct even at the fovea especially in the right eye.

A 65-year-old man complained of a recent reduction in vision of both eyes. He had been diagnosed with retinitis pigmentosa with non-recordable electroretinograms about 15 years earlier but visited the hospital irregularly. His decimal best-corrected visual acuity was 0.4 in the right eye and 0.6 in the left eye. Perimetric examinations showed a concentric constriction of the visual fields of both eyes. Ophthalmoscopy showed that the large choroidal vessels were visible which is characteristic of eyes with choroideremia, and the vessels were visible from the posterior pole to the periphery in both eyes (figure 1). We presumed this as a choroideremia case because the patient refused the genetic test. OCT (DRI-OCT, Topcon, Japan) showed a loss of the ellipsoid zone and tubulations around the fovea (figure 2).

**Figure 3:**
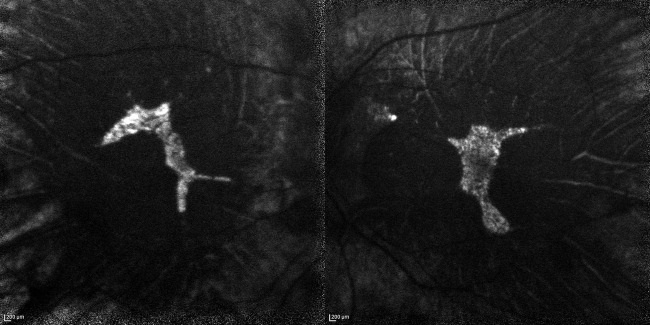
Fundus autofluorescence of both eyes. An isolated area of hyperautofluorescence can be seen in the foveal area. Part of the large choroidal vessels and transparent sclera are observed as hyperreflective tissues. FAF, fundus autofluorescence.

**Figure 4:**
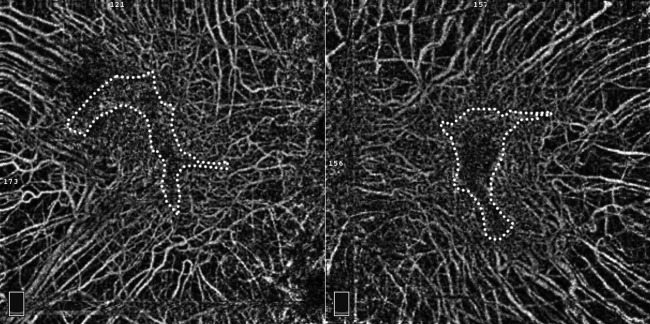
Optical coherence tomography angiography of both eyes. An 8×8 mm choroid capillary slab clearly shows the medium and large choroidal vessels surrounding the macular area. Small choroidal vessels are seen in the area (enclosed by dot lines) of the hyperautofluorescence shown in figure 3.

**Figure 5:**
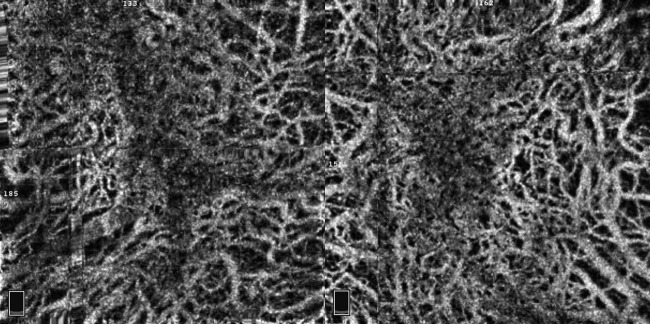
Optical coherence tomography angiography of both eyes. Choroid capillary slab of 3×3 mm shows the medium and large choroidal vessels clearly even at the foveal area. Small choroidal vessels, most likely the choriocapillaris, are densely distributed in the foveal area in the relatively wider area than the hyperautofluorescent isolated area in the FAF images (figure 3). FAF, fundus autofluorescence.

FAF (HRA2, Heidelberg, Germany) showed that the entire posterior pole was hypoautofluorescent except for an isolated area of hyperautofluorescence which included the fovea (figure 3). Some of the larger choroidal vessels were hyperreflective. The choroid capillary slab of the OCTA (RTVue XR Avanti, Optoview, Fremont, CA) images showed the medium and large choroidal vessels clearly in the 8×8 mm size macular images (figure 4). The small vessels were observed in a relatively wider area than the hyperautofluorescent area in the FAF images. These smaller vessels, probably the choriocapillaris, were seen, especially in the 3×3 mm OCTA images (figure 5).

## Discussion

Choroideremia is an X linked inherited disease caused by a deficiency of the Rab escort protein 1 (REP1) encoded by the *CHM* gene. Although it is generally believed that it is not possible to treat choroideremia by conventional therapy, a clinical trial of gene therapy using a viral vector for REP1 has been reported to be relatively successful.^1^ Choroideremia is associated with a degeneration of the RPE and choriocapillaris throughout the fundus.^2–6^ However, it is unclear whether the RPE or choriocapillaris is the first structure to be altered.

OCT is a useful method because it can non-invasively examine the morphology of the retina and choroid. Higher resolution images can be obtained for cross-sectional images after averaging many images to increase the signal-to-noise ratio. However, it is not the best method to assess the extent of the lesion such as in eyes with choroideremia. FAF, a relatively new method, can evaluate the functionality of the RPE by the changes in the fluorescence and can examine a wide area of the fundus. However, there are at least two weaknesses for assessing choroideremia by FAF. The first is that the fluorescent values from FAF are not absolute but relative values. Although FAF showed hypoautofluorescence at the periphery and hyperautofluorescence at the foveal area in our patient, it might also be iso-fluorecent and comparable to that of a normal RPE. Another disadvantage is that FAF cannot evaluate the normality of the choriocapillaris. As mentioned, choroideremia is characterised by a degeneration of choriocapillaris and RPE, but it is important to know whether the abnormalities of the choriocapillaris or the RPE are the major cause of the choroideremia disease process.

OCTA cannot image the choroidal vasculature in normal persons because of the RPE, and there are only a few studies on the choroidal blood pattern using OCTA because the vessels are not visible in normal individuals.^7^ ^8^ However, Spaide *et al*^9^ were able to obtain images of the choroidal vessels by OCTA in eyes with RPE atrophy and discussed the potential artefacts in the OCTA images. Jain *et al*^10^ reported that the choriocapillaris in eyes with choroideremia was widely damaged in their OCTA study. In our patient, the large choroidal vessels were observed ophthalmoscopically in the area of the RPE atrophy which may indicate an atrophy of the choriocapillaris as Jain *et al* described. In contrast, the smaller choroidal vessels, probably the choriocapillaris, in the area of the isolated lesion were more visible than the medium and large choroidal vessels in our case. Generally, the choriocapillaris is not clearly seen in the OCTA images even in the capillary slab of the choroid of normal persons because of the light attenuation by the RPE. This suggests that OCTA can evaluate the state of the choriocapillaris mainly in cases with RPE damage.

Although this finding from a case report cannot be generalised, the OCTA images in our patient showed the choriocapillaris in a relatively wider area than the hyperautofluorescent isolated area in the FAF images. This suggests that the RPE atrophy occurred before the choriocapillaris atrophy in this eye with choroideremia although the choriocapillaris might be damaged to some degree. Xue *et al*^11^ reported that RPE loss is the primary cause of choroideremia in their study of the relationship between outer retina/choroid using OCT and RPE atrophy using FAF. Their study also supported our findings. Further studies of more cases are needed to determine the exact course of the morphological changes in the eyes with choroideremia.

Our FAF and OCTA results suggest that the RPE disorder developed before the choriocapillaris atrophy in this case of presumed choroideremia. The findings indicate that multimodal imaging, including FAF and OCTA, can provide new information on the morphology and function of the retina and choroid in different diseases.

**Learning points**

- Although choroideremia is associated with a degeneration of the RPE and choriocapillaris throughout the fundus due to a deficiency of the Rab escort protein 1, it is unclear whether the RPE or choriocapillaris is the first structure to be altered.
- FAF can evaluate the functionality of the RPE by the changes in the fluorescence. OCTA can detect the retinal and choroidal blood vessel patterns even in the choriocapillaris slab. Since RPE is supplied by the choriocaplillaris generally, the abnormal areas in RPE should be identical between images of FAF and OCTA.
- In the current study, we evaluated RPE and choriocapillaris damage using FAF and OCTA in choroideremia case with preserved central vision. Hypoautofluorescence except for an isolated area of hyperautofluorescence which included the fovea was seen in FAF. Medium and large choroidal vessels were clearly seen throughout the fundus in OCTA. OCTA also showed the choriocapillaris in a relatively wider area than the hyperautofluorescent isolated area on FAF. It was possible to identify RPE atrophy before the choriocapillaris atrophy in choroideremia.
- The multimodal imaging, including FAF and OCTA, can provide new information on the morphology and function of the retina and choroid in different diseases.
